# Factors associated with parasympathetic activation following exercise in patients with rheumatoid arthritis: a cross-sectional study

**DOI:** 10.1186/s12872-016-0264-9

**Published:** 2016-05-10

**Authors:** Ahmad Osailan, George S. Metsios, Peter C. Rouse, Nikos Ntoumanis, Joan L. Duda, George D. Kitas, Jet J. C. S. Veldhuijzen van Zanten

**Affiliations:** School of Sport, Exercise and Rehabilitation Sciences, University of Birmingham, Birmingham, B15 2TT UK; Department of Rheumatology, Dudley Group NHS Foundation Trust, Dudley, UK; Department of Physical Activity Exercise and Health, University of Wolverhampton, Walsall, West Midlands UK; Department for Health, University of Bath, Bath, UK; School of Psychology & Speech Pathology, Curtin University, Perth, Australia

**Keywords:** Rheumatoid arthritis, Parasympathetic function, Autonomic function, Cardiovascular risk, Exercise testing

## Abstract

**Background:**

Patients with rheumatoid arthritis (RA) have an increased risk for cardiovascular disease (CVD) with poor parasympathetic function being implicated as an underlying factor. Factors related to parasympathetic function, commonly assessed by heart rate recovery (HRR) following maximal exercise, are currently not known in RA. We aimed to explore the association between HRR with CVD risk factors, inflammatory markers, and wellbeing in patients with RA.

**Methods:**

Ninety-six RA patients (54.4 ± 12.6 years, 68 % women) completed a treadmill exercise test, during which heart rate (HR) was monitored. HRR1 and HRR2 were defined as the absolute change from HR peak to HRR 1 min post HR peak and 2 min post HR peak, respectively. Cardiorespiratory fitness, CVD risk factors, and serological markers of inflammation were measured in all patients. The Framingham Risk Score (FRS) was used as an assessment of global risk for CVD events, and wellbeing was assessed by questionnaires.

**Results:**

Mean HRR1 and HRR2 were 29.1 ± 13.2 bpm and 46.4 ± 15.3 bpm, respectively. CVD risk factors as well as most inflammatory markers and measures of wellbeing were inversely correlated with HRR1 and HRR2. Multivariate regression analyses revealed that 27.9 % of the variance in HRR1 and 37.9 % of the variance in HRR2 was explained collectively by CVD risk factors, measures of inflammation, and wellbeing (*p* = 0.009, *p* = 0.001 respectively), however no individual measure was independently associated with HRR1 or HRR2.

**Conclusion:**

Parasympathetic activation was associated with overall CVD risk, arthritis-related burden and wellbeing in patients with RA.

**Trial Registration:**

[Exercise, cardiovascular disease and rheumatoid arthritis, ISRCTN04121489]

## Background

Rheumatoid arthritis (RA) is an autoimmune disease characterized by chronic systemic inflammation affecting approximately 1 % of the general population [1]. The main symptoms are pain, stiffness and swelling of joints. However, there are also extra-articular aspects in RA, such as an increased risk for cardiovascular disease (CVD), which causes more than 40 % of the deaths in RA [2]. Although the mechanisms for the increased risk for developing CVD in RA remain to be elucidated, several risk factors for CVD are prevalent in RA, such as hypertension [3] dyslipidemia [4], physical inactivity [5], inflammation [6]. This increased prevalence of CVD risk factors has been associated with abnormalities in vascular function and morphology [7], which could lead to CVD and even CVD-related death [8]. More recently, in a systematic review by Adlan et al. [9], autonomic dysfunction has been suggested as another factor which contributes to the CVD death risk in RA.

The autonomic nervous system (ANS) is divided into the sympathetic and the parasympathetic system, which, in a healthy state, work together to maintain cardiovascular autonomic balance. Autonomic dysfunction, an increased activity of the sympathetic tone and less parasympathetic tone at rest [10], has been associated with increased risk of CVD [11] and all-cause mortality [12]. Inflammation has been related to autonomic dysfunction in clinical [13], and healthy populations [14]. However, little is known about the factors that contribute to autonomic dysfunction in a population with a high long-term inflammatory burden, such as patients with RA [15].

A common method to assess autonomic function, and in particular parasympathetic tone, is heart rate recovery (HRR). The rapid fall in heart rate immediately following an exercise tolerance test (ETT) is suggested to represent reactivation of the parasympathetic tone [16]. HRR is a predictor of CVD and all-cause mortality [16], and has been associated with inflammation levels in a healthy population [14]. HRR is commonly assessed one or two minutes post exercise, which is thought to reflect the parasympathetic activity and withdrawal of sympathetic activity, respectively [17]. Even though autonomic function has been explored in RA using different methods [15, 18–40], to our knowledge, HRR following exercise has recently been investigated in a pilot study involving RA and Juvenile idiopathic RA [41]. However, the study was looking at the improvement post exercise intervention and there was no investigation over factors associated with HRR. Therefore, the aim of this cross-sectional study was to assess HRR post ETT and explore factors associated with HRR in RA. It was hypothesized that HRR is associated with markers of CVD risk, as well as markers of inflammation and wellbeing.

## Methods

### Study population

Ninety-six RA patients (fulfilling the revised American College of Rheumatology criteria [42]), were recruited from outpatient clinics of the Dudley Group NHS Foundation Trust, UK from October 2011 to 2014 to participate in an exercise intervention study (Trial registration number: ISRCTN04121489). Exclusion criteria were: fibromyalgia, recent joint surgery (in the preceding six months), comorbidity incompatible with exercise as per American College of Sports Medicine (ACSM) guidelines [43], atrial fibrillation, and established CVD. Ethical approval was obtained by the National Research Ethics Service and all patients provided written informed consent prior to participation.

### Protocol

Participants were invited to visit the research laboratory on two different occasions. During visit one, a fasted blood sample was taken and questionnaires were given. During visit two, brachial blood pressure was taken using electronic sphygmomanometer (Datascope Accutor, Mahwah, NJ, USA) while the patient was seated. Height was measured to the nearest 0.5 cm using a standard height measure (Seca 214 Road Rod), weight and BMI were measured using a Tanita BC 418 MA Segmental Body Composition Analyser (Tanita Corporation, Tokyo, Japan). Subsequently, the patients were fitted with an appropriate size mask to cover the nose and mouth securely for the purpose of inspired and expired gas analysis, and electrocardiography (ECG) (12-channel ECG custo cardio 200, custo med, Liepzig, Germany) electrodes were attached. Resting heart rate and volumes of O_2_ consumption were recorded for two minutes while seated, followed by an ETT and six minutes post ETT recovery period.

### Exercise Tolerance Test (ETT)

ETT was performed on a treadmill (HP Cosmos Mercury, Nussdoerf-Traunstien, Germany). All patients performed an individualized treadmill ETT which was modified according to their fitness and physical abilities [44]. The starting speed of the test was set based on the patient’s preference (approximately three mph) and 0 % inclination. Thereafter, the speed was gradually increased until a maximum brisk walking was reached, again based on the patient’s ability.

After two minutes warming up at the preferred speed, the test started. The inclination increased by one percent every minute while keeping the speed constant. Breath by breath gas analyses (Metalyzer 3B, Cortex, Liepzig, Germany) were recorded throughout the exercise task, which was used to calculate peak volume of O_2_ uptake (VO_2_ peak). ECG was recorded throughout the exercise task and recovery period. The test was terminated if patients reached volitional exhaustion, or any of the relative or absolute contraindications of ACSM’s guidelines [43] were met. Following the termination of the test, patients were asked to rest on a chair for a minimum of six minutes recovery while blood pressure was measured every two minutes.

### Outcome measures

#### Heart rate recovery

HRR was measured at 1 and 2 min following peak heart rate during exercise. Heart rate recovery 1 min (HRR1) was defined as the absolute change from peak heart rate to heart rate 1 min post peak heart rate (HRR1 = peak heart rate – heart rate at 1 min post peak heart rate). Similarly, heart rate recovery 2 min (HRR2) was calculated as the absolute change from peak heart rate to heart rate 2 min post peak heart rate. Peak heart rate was identified as the maximum heart rate during the exercise protocol.

### VO_2_ peak

Aerobic capacity (VO_2_ peak) was measured during treadmill ETT via a breath by breath gas analyser. The inspired and expired gases data from the analyser were averaged every 2 s. To avoid spikes and fluctuations of the VO_2_ ml/min readings, these data were smoothened by taking the average of VO_2_ every 28 s (taking the average of 14 readings of VO_2_ ml/min). VO_2_ peak was defined as the highest VO_2_ during the test and was expressed as ml/min/kg.

### Serological assessments

The blood samples were analysed for serological risk factors for CVD: total cholesterol, high density lipoprotein (HDL), low density lipoprotein (LDL), and triglycerides. Homeostasis models assessment (HOMA) was utilized to assess insulin resistance [45]. Inflammatory markers were assessed including erythrocyte sedimentation rate (ESR), high sensitivity C-reactive protein (hsCRP), fibrinogen, and white blood cells (WBC). Analyses were carried out using routine laboratory procedures in the hospital’s laboratory.

### Global cardiovascular risk

In order to measure the probability of occurrence of cardiovascular events within 10 years period, the Framingham risk score was utilized [46].

### Measures of wellbeing

Functional ability, psychological and general wellbeing were assessed using health assessment questionnaire (HAQ) [47], hospital anxiety and depression scale (HADS) [48], subjective vitality scale [49], multidimensional assessment of fatigue (MAF) [50], and the European quality of life questionnaire (EuroQol) [51].

### Statistical analysis

Statistical analysis was performed using SPSS20 (Chicago, IL, USA). Kolmogorov-Smirnov test was used to test the normality of all variables. Log-transformation was performed for non-normally distributed variables (BMI, triglycerides, LDL, hsCRP, ESR, HOMA, FRS, HADS depression, EuroQol). All normally distributed variables were presented as mean and standard deviation, whereas, non-normally distributed variables were presented as median and interquartile range. To assess the relationship between HRR at both time points and other normally distributed variables, bivariate correlation (correcting for gender as a possible confounder) using Pearsons moment. Bivariate correlation was performed on normally distributed variables and after log transformation for the skewed variables. To assess factors associated with HRR at both time points, two multivariate linear regression were used using enter method (correcting for gender) where HRR (HRR1 and HRR2 separately) was the dependent variable, while the independent variables were all the variables that came out significantly associated with HRR1 and HRR2 separately in our correlation analysis. The level of significance for all analyses was set at .05.

## Results

### Patient characteristics

Demographic characteristics as well as CVD risk, markers of inflammation and wellbeing of the 96 RA patients (66 female, 30 male, disease duration 7.9 ± 9.1 years) are presented in Table 1. The most common comorbidities were hypertension (33 %) and diabetes (8 %), and 9 % were current smokers whereas 44 % of the patients were previous smokers. The types of medication used included: Methotrexate (74 %), other non-biologic disease modifying anti-rheumatic drugs (DMARDs) (52 %), anti-tumor necrosis factor (12 %), other biologic DMARDs (3 %), prednisolone (16 %), non-steroidal anti-inflammatory drug (35 %), analgesics (37 %), cholesterol-lowering (27 %), and anti-hypertensive (24 %). Patients were requested to maintain their course of treatment during the study. The results from the exercise tests, including HRR1 and HRR2, are presented in Table 2. On average, the overall fitness level of the participants was low as indicated by their mean VO_2_ max 20.6 ± 5.1 ml/min/kg [52].

**Table 1:** Demographic characteristics, CVD risk, inflammatory markers, and measures of wellbeing

Characteristics	Value
Cardiovascular risk factors	
Age (years)	54.4 ± 12.6
Height (m)	1.7 ± .09
Weight (Kg)	79.3 ± 18.5
BMI (Kg/m^2^)	27.8 (23.9–31)
Heart rate rest (bpm)	79.7 ± 12.5
Resting SBP (mmHg)	134.2 ± 17
Resting DBP (mmHg)	81 ± 9.7
Total cholesterol (mmol/L)	5.1 ± 1.05
Triglycerides (mmol/L)	1.1 (0.8–1.4)
HDL (mmol/L)	1.4 ± 0.38
LDL (mmol/L)	3.1 (2.5–3.6)
HOMA	1.5 (0.94–2.05)
Framingham risk score	7 (4–13)
Inflammatory markers	
WBC (10^9^/ litre)	6.8 ± 2.4
Fibrinogen (g/L)	4.7 ± 1.1
HsCRP (mg/L)	4.6 (1.6–8.8)
ESR (mm/hr)	10 (5–21.5)
Wellbeing	
Vitality	4.1 ± 1.6
HADS anxiety	6.7 ± 4.2
HADS depression	4 (2–7)
Global Fatigue	25.8 ± 11.8
EuroQol	0.7 (0.6–0.8)

Values are presented as means ± standard deviation, or median (25th to 75th percentile values) as appropriate. BMI, body mass index; SBP, systolic blood pressure; DBP, diastolic blood pressure; HDL, high density lipoprotein; LDL, low density lipoprotein; WBC, white blood cells; HsCRP, high sensitivity C-reactive protein; ESR, erythrocyte sedimentation rate; HOMA, homeostasis model assessment insulin resistance; Vitality, score ranges 0 (low vitality) – 6 (high vitality); HADS, hospital anxiety and depression scale (0–21); Global fatigue, score ranges from 0 (no fatigue) – 50 (severe fatigue) EuroQol, European assessment for quality of life

**Table 2:** Exercise related variables

Variable	Value (Mean ± SD)
Heart rate peak (bpm)	153.4 ± 19.7
VO_2_ peak (ml/min/kg)	20.6 ± 5.1
RER (VCO_2_/VO_2_)	1.2 ± 0.1
HRR1 (bpm)	29.1 ± 13.2
HRR2 (bpm)	46.4 ± 15.3

VO_2_ peak, highest volume of oxygen; RER, respiratory exchange ratio; HRR1, the difference between HR peak and heart rate 1 min post HR peak; HRR2, the difference between HR peak and heart rate 2 min post HR peak

### Correlations

Correlational analyses were conducted to explore the associations between HRR and risk factors for CVD and wellbeing. With regards to CVD risk factors, results revealed that HRR1 and HRR2 were inversely associated with age and triglycerides, whereas resting SBP was only inversely associated with HRR2 (Table 3). HRR2 was inversely associated with HOMA (*r* (87) = − .27, *p* = .009). VO_2_ peak was positively associated with HRR1 and HRR2 (*r* (93) = .33, *p* = .001 and *r* (93) = .41, *p* < .001, respectively). HRR1 and HRR2 were both inversely related to FRS. WBC, Fibrinogen, hsCRP, and ESR were all inversely associated with HRR1, and, albeit somewhat weaker, with HRR2 with the exception of CRP (Table 3). With regards to measures of wellbeing, EuroQol and vitality were positively associated with HRR1 (*r* (90) = .26, *p* = .01 and *r* (88) = .30, *p* = .004), respectively, whereas, only vitality was significantly associated with HRR2 (*r* (88) = .21, *p* = 0.05). Additional non-parametric correlational analyses (Spearman correlation) were conducted between HRR and those variables which were not normally distributed. These analyses revealed similar associations as the Pearsons correlations reported on the log-transformed data (data not reported).

**Table 3:** Correlation of HRR1 and HRR2 with CVD risk, inflammatory markers, and wellbeing

	HRR1		HRR2	
Variable	*r*	*p*	*r*	*p*
Cardiovascular risk factors				
Age	−.27	**.006**	−.44	**<.001**
BMI	−.15	.14	−.08	.43
Resting SBP	−.18	.08	−.28	**.005**
Resting DBP	−.09	.38	−.10	.33
Triglycerides	−.31	**.003**	−.27	**.008**
HOMA	−.14	.18	−.27	**.009**
VO_2_ peak	.33	**.001**	.41	**<.001**
Framingham risk score	−.29	**.005**	−.45	**<.001**
Inflammatory markers				
WBC	−.23	**.02**	−.22	**.04**
Fibrinogen	−.32	**.001**	−.27	**.009**
hsCRP	−.22	**.03**	−.15	.13
ESR	−.26	**.01**	−.21	**.03**
Wellbeing				
Vitality	.30	**.004**	.20	**.05**
HADS depression	−.18	.07	−.03	.81
HADS Anxiety	−.02	.87	.12	.21
Global fatigue	−.19	.054	−.03	.75
EuroQol	.26	**.01**	.12	.24

HADS, hospital anxiety and depression scale; EuroQol, European assessment for quality of life. All variables were corrected for gender. *p* value in bold indicate statistical significance

### Linear regression

In order to identify if the variables were independently associated with HRR, all variables which were significantly correlated with HRR were subsequently entered in multivariate linear regression analyses (see Table 4). A model (based on the correlated variables in the univariate analyses) which included age, triglycerides, VO_2_ peak, Framingham risk score, WBC, CRP, ESR, fibrinogen, vitality, and EuroQol accounted for 29.7 % of the variation in HRR1 (*F* (11, 67) = 2.57, *p* = .009, R^2^ = 0.29). However, even though almost a third of the variance in HRR1 was explained by these variables, none of the variables were independently associated with HRR1. For HRR2, using the same method, a model which included age, resting systolic blood pressure, triglycerides, HOMA, VO_2_ peak, Framingham risk score, WBC, ESR, fibrinogen and vitality accounted for 37.9 % of the variation in HRR2 (*F* (11, 65) = 3.6, *p* = .001, R^2^ = 0.37). Similar to HRR2, none of the variables were independently associated with HRR2. Data was checked for collinearity, but this revealed not to influence the data. Additionally, in a different analyses, using age as a confounding factor along with gender did not change the model presented in Table 4 (data not reported).

**Table 4:** Linear regression model for factors associated with HRR1 and HRR2

	HRR1		HRR2	
Variable	β	t (*p*)	β	t (*p*)
Cardiovascular risk factors				
Age	−0.26	−1.25 (.21)	−0.46	−1.91 (.06)
Triglycerides	−21.69	−1.48 (.14)	−6.03	−0.37 (.71)
VO_2_ peak	0.05	0.11 (.91)	0.45	−0.98 (.32)
Framingham risk score	0.09	0.01 (.99)	−2.72	−0.25 (.79)
Resting SBP^a^			−0.07	−0.69 (.48)
HOMA^a^			−2.51	−0.16 (.86)
Inflammatory markers				
WBC	−0.57	−0.66 (.51)	−0.01	−0.02 (.98)
Fibrinogen	−3.18	−1.36 (.17)	−2.24	−0.94 (.34)
hsCRP^b^	0.86	0.16 (.87)		
ESR	4.49	0.70 (.48)	6.62	0.99 (.32)
Wellbeing				
Vitality	1.27	0.98 (.32)	2.09	1.78 (.07)
EuroQol^b^	33.65	1.09 (.27)		
R2 and p-value of the model	R^2^	*p*	R^2^	*p*
	0.297	**0.009**	0.379	**0.001**

^a^Indicates variable that was not correlated with HRR1 in univariate analyses, therefore, was not included in model 1

^b^Indicates variable that was not correlated with HRR2 in univariate analyses, therefore, was not included in model 2

*p *value in bold indicate statistical significance

## Discussion

To our knowledge, this is the first study to assess the relationship between HRR and CVD risk factors, disease-related measures and indicators of wellbeing in RA patients. As expected, many CVD risk factors, inflammatory markers and some measures of wellbeing were correlated with HRR. However, multivariate analyses revealed that the variance in HRR was explained by a group of factors including CVD risk factors, inflammatory markers and some measures of wellbeing, but none of the variables tested were independently associated with HRR. This may suggest that it is the overall CVD risk and disease-related burden that contributes to changes in HRR, instead of one or several individual factors.

The current study is the first to reveal that HRR was correlated with individual CVD risk factors in RA. In line with other populations [53], an association was found between triglycerides and HRR, which is not surprising given the association between lipid metabolism and the ANS [54]. As expected based on findings from other populations [55], cardiorespiratory fitness was also correlated with HRR, a phenomenon mainly attributed to improved baroreflex sensitivity [56]. Moreover, our results revealed that age was inversely associated with HRR which is in line with some [30, 35], but not all studies [27, 31] in this field. Similar equivocal findings have been found between autonomic function and body composition [24, 27]. The variation in findings of the available studies is probably due to the differences in the ANS assessment.

To our knowledge, this is the first study to report an association between global CVD risk (FRS) and HRR in RA. The effect size of the associations between global CVD risk and HRR were greater than the effect sizes of the associations between individual risk factors and HRR. Given that FRS comprises of multiple variables, this association, together with the finding that none of the individual risk factors were independently associated with HRR in multivariate analyses, suggests that rather than individual risk factor, the overall RA- and CVD related burden is an important factor that associates with parasympathetic function.

In line with cross-sectional and experimental studies in other populations [14, 57], disease-related inflammation was inversely associated with HRR. In RA patients however, the results are equivocal. As recently reviewed, the majority of the studies do not find an association between inflammation or disease activity and autonomic function in RA, even though there are some exceptions [9]. For example, CRP [35], and leukocytes [34] were reported to be related to autonomic dysfunction, but more studies reported no such association [15, 19, 23, 24, 33]. Similar to inflammation, disease activity (DAS28) was found to be associated with autonomic function in some [20, 30, 34], but not all studies [15, 19, 23, 24, 31, 33]. Comparison between the different studies is difficult given the different methods used to assess autonomic function (i.e., heart rate variability (HRV) [36], pupillary response to light [31], Ewing test [22], and HRR as in the current study). When exploring the available studies in more detail, it is worth noting that studies that reported no association between autonomic function and inflammation used the Ewing test battery as a method of assessment [15], whereas those who did report an association measured autonomic function using HRV [35]. The Ewing test battery consists of five separate assessments with a dichotomized response to each test and the total score is the sum of all the responses [58]. HRV and HRR in contrast, use continuous heart rate readings to derive a measure of autonomic function. Therefore, it could be argued that statistically HRV and HRR are more sensitive tests for exploring associations compared to the Ewing test battery. However, without making a direct comparison between factors associated with the Ewing test battery and HRV or HRR in the same population, this suggestion remains speculative.

There were subtle differences in the associations between inflammation and HRR1 and HRR2 in the current study; CRP was associated with HRR1 which is related to parasympathetic reactivation, but not HRR2, which is reflective of sympathetic withdrawal [17]. Similarly, inducing inflammation reduced HRR1 but not HRR2 in healthy participants [14]. Interestingly, the evidence for parasympathetic dysfunction is also more evident in RA compared to sympathetic dysfunction [9]. From a theoretical standpoint, the association between inflammation and the parasympathetic system is not unexpected. This could be explained via the mechanism of cholinergic anti-inflammatory pathway, where release of inflammatory markers such as tumor necrosis factor alpha and interleukin-1 is controlled by the vagus nerve which is part of the parasympathetic system [59]. However, future research is needed to explore this association in more detail in patients with RA.

The first two minutes of HRR are the most validated [60] and commonly used method in studies, and have been suggested to be a better predictor of mortality and coronary artery disease (CAD) [61]. The subtle differences in the factors related with HRR1 and HRR could perhaps be due to the different aspects of ANS which are reflected by HRR1 and HRR2: HRR1 is known to represent activation of parasympathetic system, whereas, HRR2 represent activation of parasympathetic and withdrawal of sympathetic nervous system [62]. In the current study, SBP and insulin resistance were significantly associated with HRR2 but not HRR1. Interestingly, insulin has been found to have a stimulatory effect on the sympathetic nervous system [63], and metabolic risk factors have been more strongly related to HRR2 than HRR1 in healthy participants. However, this complex interaction between the two systems in controlling post exercise HRR still needs further clarification.

To our knowledge, this is the first study to explore associations between markers of psychological wellbeing and autonomic function in RA. This is surprising given that psychological factors have been associated with CVD and autonomic function has been implicated as an underlying pathway by which psychological wellbeing contributes towards the pathogenesis of CVD [64]. Indeed, history of anxiety and depression [65], as well as emotional stress [66], have been associated with lower parasympathetic activity. In the current study, even though no significant relationship was found between either depression or anxiety and HRR, vitality and quality of life (EuroQol) were related to HRR. This may suggest that maintaining a good quality of life and the perception of feeling energized and capable of doing tasks may favor a balanced activity of the ANS.

There are number of limitations in this study. The cross-sectional design does not allow for the assessment of causality between variables. The current study did not have a comparison group, however, as the aim of the current study was to explore factors associated with parasympathetic activity in RA, the focus was specifically on RA patients. Without a control group, a direct comparison between parasympathetic activity in RA and other populations is not possible. Comparing HRR in this well-controlled cohort of RA patients from this study to HRR reported in healthy participants in other studies [67, 68], reveal similarities in parasympathetic function. Caution should of course be taken when comparing different studies as variations in the methods are likely to influence the findings. Therefore, future studies should examine differences between HRR in RA and other population as well as exploring factors associated with HRR in other population. For ethical reasons, the patients did not discontinue their medication prior to the assessments. As is common in this population, the patients included in this study were on a mix of medications, which means that we did not have an appropriate statistical power to explore the influence of medication on HRR. Further research should investigate the potential influence of medication on HRR in patients with RA. The disease activity of most of the RA patients involved in this study was well controlled; therefore, it is difficult to generalize these findings to RA patients with higher disease activity. It is possible that patients were not able to exercise to their maximum cardiorespiratory ability due to problems with their joints, which could have influenced their HRR [17]. However, based on respiratory exchange ratios (RER) which reflect the amount of energy metabolized to provide energy, we are confident that most of the participants in our study were able to exercise until exhaustion. Future research should also explore the association between CVD risk, inflammation, and wellbeing with other measures of ANS, such as HRV.

## Conclusions

In conclusion, the results of this study showed that CVD risk, disease related inflammations, and wellbeing associate with HRR in RA. As there is ample evidence that HRR is related to CVD mortality in other populations [16], further research should explore if HRR is also predictive of hard CVD endpoints in RA. In addition, given that exercise interventions can reduce CVD risk in RA [69], and improve parasympathetic reactivation [70], further research should also examine the effects of an exercise intervention on HRR in this population. The results of this study suggest that reducing CVD risk in RA is likely to require interventions to improve classical risk factors, inflammation, physical fitness, and psychological well-being. HRR could be used as an overall marker of patients at risk of developing CVD, therefore, can be used as a marker to see whether an intervention is effective in this cohort. However, this will need further research to investigate the effectiveness of an intervention using HRR.

### Availability of data and materials

This data forms part of a larger ongoing study, it is therefore not possible to share the data at this stage.

## Abbreviations

ACSM: American college of sports medicine
ANS: autonomic nervous system
BMI: body mass index
CRP: C-reactive protein
CVD: cardiovascular disease
DMARDs: disease modifying anti-rheumatic drugs
ECG: electrocardiograph
ESR: erythrocyte sedimentation rate
ETT: exercise tolerance test
EuroQol: European quality of life questionnaire
FRS: Framingham risk score
HADS: hospital anxiety and depression scale
HAQ: health assessment questionnaire
HDL: high density lipoprotein
HOMA: hemostasis model assessment
HR: heart rate
HRR: heart rate recovery
HRV: heart rate variability
HsCRP: high sensitivity C-reactive protein
LDL: low density lipoprotein
MAF: multidimensional assessment of fatigue
RA: rheumatoid arthritis
VO_2_: volume of oxygen
WBC: white blood cells
